# Thermosensitive Mucoadhesive Intranasal In Situ Gel of Risperidone for Nose-to-Brain Targeting: Physiochemical and Pharmacokinetics Study

**DOI:** 10.3390/ph18060871

**Published:** 2025-06-11

**Authors:** Mahendra Singh, Sanjay Kumar, Ramachandran Vinayagam, Ramachandran Samivel

**Affiliations:** 1 Department of Biotechnology, College of Life and Applied Sciences, Yeungnam University, Gyeongsan 38541, Republic of Korea; m.singh2685@gmail.com; 2 Formulation Research and Development, Naari Pharma Private Limited, Sec-5, Rudrapur 263153, India; sanjay1535kumar@gmail.com; 3 Cornea Research Chair, Department of Optometry, College of Applied Medical Sciences, King Saud University, Riyadh 11433, Saudi Arabia

**Keywords:** CNS disorders, diffusion, thermosensitive in situ gels, nose-to-brain targeting, non-invasive, blood–brain barrier

## Abstract

**Background/Objectives:** Non-invasive central nervous system (CNS) therapies are limited by complex mechanisms and the blood–brain barrier, but nasal delivery offers a promising alternative. The study planned to develop a non-invasive in situ intranasal mucoadhesive thermosensitive gel to deliver CNS-active risperidone via nose-to-brain targeting. Risperidone, a second-generation antipsychotic, has shown efficacy in managing both psychotic and mood-related symptoms. The mucoadhesive gel formulations help to prolong the residence time at the nasal absorption site, thereby facilitating the uptake of the drug. **Methods:** The poloxamer 407 (18.0% *w*/*v*), HPMC K100M and K15M (0.3–0.5% *w*/*v*), and benzalkonium chloride (0.1% *v*/*v*) were used as thermosensitive polymers, a mucoadhesive agent, and a preservative, respectively, for the development of in situ thermosensitive gel. The developed formulations were evaluated for various parameters. **Results:** The pH, gelation temperature, gelation time, and drug content were found to be 6.20 ± 0.026–6.37 ± 0.015, 34.25 ± 1.10–37.50 ± 1.05 °C, 1.65 ± 0.30–2.50 ± 0.55 min, and 95.58 ± 2.37–98.03 ± 1.68%, respectively. Furthermore, the optimized F3 formulation showed satisfactory gelling capacity (9.52 ± 0.513 h) and an acceptable mucoadhesive strength (1110.65 ± 6.87 dyne/cm^2^). Diffusion of the drug through the egg membrane depended on the formulation’s viscosity, and the F3 formulation explained the first-order release kinetics, indicating concentration-dependent drug diffusion with *n* < 0.45 (0.398) value, indicating the Fickian-diffusion (diffusional case I). The pharmacokinetic study was performed with male Wistar albino rats, and the F3 in situ thermosensitive risperidone gel confirmed significantly (*p* < 0.05) ~5.4 times higher brain AUC_0–∞_ when administered intranasally compared to the oral solution. **Conclusions:** Based on physicochemical, in vitro, and in vivo parameters, it can be concluded that in situ thermosensitive gel is suitable for administration of risperidone through the nasal route and can enhance patient compliance through ease of application and with less repeated administration.

## 1. Introduction

The risperidone (RIS) is 3-(2-(4-(6-fluorobenzo[d]isoxazol-3-yl) piperidin-1-yl) ethyl)-2-methyl-6,7,8,9-tetrahydropyrido [1,2-a] pyrimidin-4-3. Risperidone exists as a white and crystalline powder. It is a benzisoxazole class approved antipsychotic drug, which is marketed as an oral liquid, disintegrating tablet, and regular tablet [1]. This effective medicine is generally advised to treat bipolar mania and schizophrenia and has a poor dissolution rate due to being practically insoluble in water [2]. The water solubility of RIS at 25 and 37 °C has been reported as 2.8 and 5 μg mL^−1^, respectively. Poor water solubility and extensive first hepatic pass metabolism of RIS are the main obstacles to the poor in vivo absorption/bioavailability of RIS [1].

Nose-to-brain (N2B) delivery has appeared as a favorable alternative route to conventional drug delivery for treating central nervous system (CNS) disorders [3,4]. By bypassing the blood–brain barriers (BBBs), N2B offers a direct, non-invasive route for therapeutic agents to reach the brain [5,6]. This targeted delivery mechanism uses the olfactory and trigeminal nerve pathways, helping to achieve rapid and localized drug concentrations in the brain [7,8].

With the continuous increase in the aging population, the occurrence of neurological disorders has steadily increased, and becoming the most common, burdensome, and underserved disease [9]. By 2020, there was a substantial rise in the number of people facing anxiety and depression due to the COVID-19 pandemic. According to preliminary estimations, there has been a 26% upsurge in anxiety disorders and a 28% increase in severe depressive disorders in just 1 year [10]. The treatment of CNS conditions, such as depression, anxiety, Alzheimer’s disease, Parkinson’s disease, epilepsy, brain tumors, and stroke, is hindered by the BBBs [11,12]. These complicated BBBs limit the access of maximum drugs into the brain, restricting therapeutic efficacy, and require high systemic doses that can lead to systemic toxicity. N2B delivery offers an attractive strategy to circumvent these limitations by delivering drugs directly to the brain via the nasal cavity.

Factors influencing N2B delivery include mucociliary clearance, enzymatic degradation (such as peptidases and cytochrome P450 enzymes), molecular weight and lipophilicity, and formulation factors (particle size, viscosity, and pH) [4,13,14].

To overcome these factors, optimizing drug formulations is crucial for maximizing N2B delivery of drug(s). Several strategies have been developed to enhance drug absorption, prolong residence time, and protect drug(s) from enzymatic degradation. Nanoparticles (NPs), including liposomes, polymeric NPs, and solid lipid NPs, can encapsulate drugs, protecting them from degradation and enhancing their transport across the nasal epithelium. NPs can also be functionalized with targeting ligands to improve specificity for certain brain regions [15,16]. Microemulsions are thermodynamically stable, transparent dispersions of oil, water, and surfactants that can enhance drug solubility and permeability across the nasal mucosa [17,18]. Penetration enhancers, such as surfactants, cyclodextrins, and chitosan, can temporarily disrupt the tight junctions between epithelial cells of the BBB, facilitating drug absorption [19].

Furthermore, Sipos and colleagues prepared risperidone-loaded nasal thermosensitive polymeric micelles using quality by design formulations with Pluronic^®^ F-108 and Pluronic^®^ F-127 [20]. They observed a rapid, burst-like in vitro drug release profile, with a quick and high permeation rate at nasal conditions. In addition, Abdallah and colleagues developed risperidone-loaded glycethosomal in situ gels using Box–Behnken design for the treatment of schizophrenia-induced rats via the intranasal route [21]. They utilized poloxamer 407 and HPMC K4M gel in a ratio of 4:1 gel/glycethosomes for preparing low viscosity and high spreadability with acceptable pH, gel strength, and mucoadhesive strength ranges. The pharmacokinetic analysis of the optimized gel confirmed eight-fold and three-fold improvements in drug bioavailability against the control gel and the marketed tablet, respectively.

Hydroxypropyl Methylcellulose (HPMC) is a commonly used ingredient in various industries, including pharmaceuticals and cosmetics. It is a versatile compound that offers a wide range of benefits, such as thickening, binding, gelling, bioadhesion, sustained/controlled release, and film-forming properties [22]. HPMC is derived from cellulose ether with multiple hydroxyl groups. HPMC itself has thermogelling properties. Thus, for intranasal delivery, HPMC is usually added as a mucoadhesive or thickening agent and combined with a thermoresponsive polymer [23,24]. However, there are different grades of HPMC available based on viscosities and molecular weights, for instance, HPMC K15M, HPMC K100M, etc. HPMC K15M is a low molecular weight and viscosity polymer, while HPMC K100M is a high molecular weight and viscosity polymer [25]. HPMC K15M offers faster gelation, easier administration, and faster drug release. HPMC K100M forms a more rigid and robust gel, potentially leading to prolonged drug release and longer residence time in the nasal cavity. Hence, HPMC K100M might have better mucoadhesive properties than HPMC K15M polymer. For sustained release and prolonged residence time, HPMC K100M is often preferred. If ease of administration and faster release are the primary goals, HPMC K15M may be a better choice. Often, researchers explore combinations of both grades to achieve an optimal balance of properties.

Furthermore, chitosan is a cationic polymer with multifaceted benefits. It is a naturally derived polysaccharide obtained from the deacetylation of chitin, which offers a unique set of properties that make it attractive for nose-to-brain drug delivery [26,27]. The properties of chitosan can vary depending on the source and degree of deacetylation. Due to the presence of a positive charge in chitosan, it can easily interact with the negatively charged mucin layer of the nasal mucosa, resulting in strong mucoadhesion and prolonged residence time [28,29]. Chitosan can transiently open tight junctions between epithelial cells, enhancing drug permeation across the nasal membrane and facilitating access to the olfactory and trigeminal pathways. It is generally considered safe, biocompatible, and biodegradable, minimizing potential toxicity concerns [30,31]. It is a pH-sensitive gelling agent and soluble in acidic solutions [32]. Upon contact with the relatively neutral pH of the nasal cavity, it can precipitate. Also, chitosan poses some other limitations; for instance, it is not soluble in neutral or alkaline pHs, so it requires acidic conditions to make a gel [32,33]. In addition, at high concentrations, chitosan can cause nasal irritation in some individuals [34]. Hence, among chitosan and HPMC polymers, HPMC can be a good option for developing an in situ thermosensitive gel due to solubility issues and irritation properties of chitosan.

In situ thermosensitive gel formulations are liquid at room temperature but undergo a phase transition to form a gel upon contact with the nasal mucosa, which extends the drug residence time in the nasal cavity and improves absorption [35,36,37]. With the benefit of localized and prolonged release of therapeutic drugs directly to the brain, in situ thermosensitive gels have become a viable drug delivery method for neuroprotection. These thermosensitive gels are usually made of polymers such as poloxamer, poly (N-isopropylacrylamide, polyethylene glycol polyester, chitosan, etc. [38,39,40]. Among them, poloxamer can improve the bioavailability of drugs due to its amphiphilicity and the ability to self-assemble to form micelles [41]. These polymer solutions are liquids at room temperature but change from a liquid to a gel when they reach body temperature. This makes it simple to administer at the intended location, ensuring that a drug depot may be constructed. This targeted delivery maximizes the concentration of the neuroprotective drug at the site of damage or disease while minimizing systemic exposure and potential negative effects.

The goal of the task is to develop a thermosensitive in situ gel dosage form that will improve the issue of drug short residence duration in the nasal cavity and drug absorption in the brain via nose-to-brain delivery. Therefore, the current study aimed to develop an intranasal in situ thermosensitive gel for risperidone administration targeting the nose-to-brain pathway and to further investigate the pharmacokinetics of the drug in rats. Additionally, various physicochemical parameters, such as gelation time, gelation capacity, mucoadhesive strength, and viscosity at different temperatures, were evaluated.

## 2. Results

### 2.1. Preparation of RIS In Situ Thermosensitive Gel

Four in situ gel formulations with drugs comprising polymers such as poloxamer 407, HPMC K100M, and HPMC K15M and other ingredients were prepared. In all formulations, the concentrations of poloxamer 407 and other ingredients were kept constant, except for HPMC (Table 1). HPMC was used as a mucoadhesive agent, poloxamer 407 was employed as a thermosensitive polymer, and benzalkonium was added as a preservative.

### 2.2. Physical Appearance and pH

The prepared in situ thermosensitive gels were found to be homogeneous, transparent, and clear in appearance. The pH of the developed formulations was found to be in the range of 6.20 ± 0.026–6.37 ± 0.015, as shown in Table 2.

### 2.3. Gelation Temperature and Gelation Time

The gelation temperature and gelation time for all formulations were found in the range of 34.25–37.50 °C and 1.65–2.50 min, respectively (Table 2). It was found that as the concentration of HPMC K100M and K15M rises, a decrease in gelation temperature and gelation time was observed (Table 1 and Table 2). The time of the sol-to-gel transition was observed to be slightly higher in formulations F1 and F2 than in formulations F3 and F4. The plausible reason for this behavior may be the low concentration of HPMC (0.3% *w*/*v*) in formulations F1 and F2 as compared to formulations F3 and F4, which contained HPMC in a concentration (0.5% *w*/*v*).

### 2.4. Drug Content of the Prepared In Situ Thermosensitive Gel

The final drug content was observed in the range of 95.58–98.03% (Table 2).

### 2.5. Viscosity of In Situ Thermosensitive Gel

**Figure 1:**
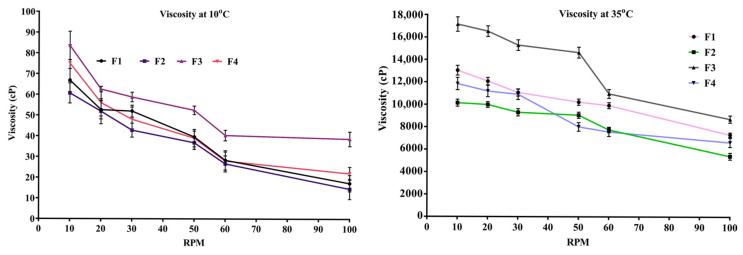
Graphical representation of viscosity at different temperatures and RPM.

The results of the study, shown in Figure 1, reveal that the viscosity of the formulations (F1–F4) changed significantly due to a change in the grade or concentration of HPMC. Viscosity was found in the range of 5410 ± 304.91 cps to 17,160 ± 646.87 cps at a temperature of 35 °C, while it was observed between 14.54 ± 4.91 cps and 83.54 ± 6.87 cps at 10 °C temperature. It was observed that as the shear speed increased, the viscosity of the formulation decreased, as shown in Figure 1. The viscosity of F3 in situ gel at 10 and 100 rpm was found to be 83.54 ± 6.87 cps and 38.65 ± 3.49 cps at 10 °C and 17,160 ± 646.87 cps and 8726 ± 323.49 cps at 35 °C, respectively.

### 2.6. Gelling Capacity and Spreadability

The results of the gelling capacity are shown in Table 3. The gelling capacity of the in situ gel depends on poloxamer 407. The gelling capacity of prepared formulations was found to be highly adequate for 9–10 h (Table 3).

Spreadability of an intranasal in situ gel is also an important parameter due to the ease of application and ease of spreading on the nasal mucosa without leakage after administration. The spreadability of formulated gels was observed in the range from 18.24 ± 2.15 to 33.66 ± 2.08 g.cm/s The observed data in all the tested in situ gel formulations have satisfactory spreadability and could be suitable for nasal application.

### 2.7. In Vitro Mucoadhesive Strength

**Figure 2:**
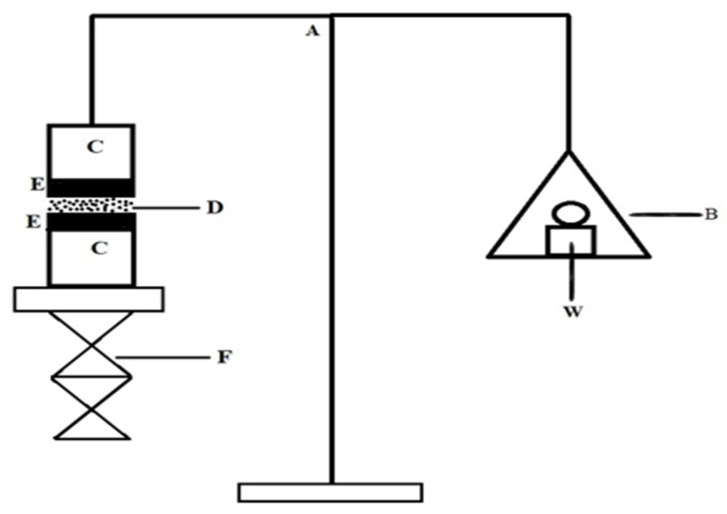
Graphical representation of analytical balance for measuring mucoadhesive strength: (A) analytical balance; (B) weighing pan; (W) weight; (C) glass vial; (D) in situ formulation; (E) nasal membrane; (F) height adjustable pan.

The mucoadhesive intensity of the gel was evaluated using an analytical balance method (Figure 2). The results of the prepared in situ gel formulations are shown in Table 3. HPMC (0.3% *w*/*v*) was used in formulations F1 and F2, whereas formulations F3 and F4 contained (0.5% *w*/*v*) HPMC as a mucoadhesive agent. The adhesive characteristics of gel formulations were observed to be enhanced with increasing HPMC content, as indicated by a study on dissociation stress and mucoadhesive strength [37].

### 2.8. In Vitro Diffusion Study

**Figure 3:**
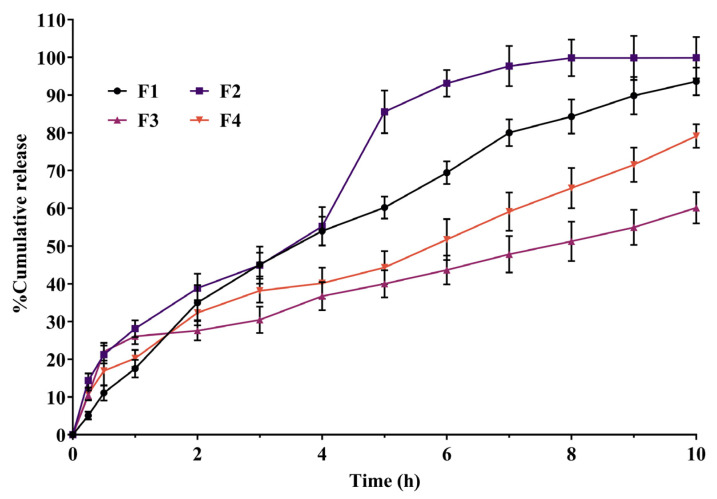
Percentage of in vitro diffusion of drug from various in situ intranasal gels.

The drug release study was performed for 10 h in a phosphate buffer at pH 6.0 to determine the therapeutic response and absorption properties of the drug from the in situ gel. The in situ gel’s drug release profile was influenced by the concentration and grade of HPMC utilized in the formulations, which acts as a mucoadhesive polymer to retard the drug release from the formulations. The release profiles of the drug are mentioned in Figure 3.

As the concentration of HPMC K100M and HPMC K15M was increased, the drug release was reduced (Figure 3). The formulation F1 initially released the drug slowly in the first hour, but it released 70% in 6 h, while the F2 formulation initially (1 h) showed faster drug release, following that, the drug was gradually released, and after 6 h, more than 90% of the drug was released. The difference in drug release pattern may be due to the grade of HPMC and the viscosity differences in the polymer.

Similarly, formulations F3 and F4 exhibited a sustained/controlled type of drug release pattern (Figure 3). The F3 formulation released only 60% of the drug in 10 h, while the F4 formulation comparatively released 79% of the drug in 10 h. However, formulation F3 is highly controlled when compared to the remaining formulations because it contains 0.5% HPMC K100M polymer. The differences in the release pattern may be due to concentration and type of HPMC as a mucoadhesive polymer.

**Figure 4:**
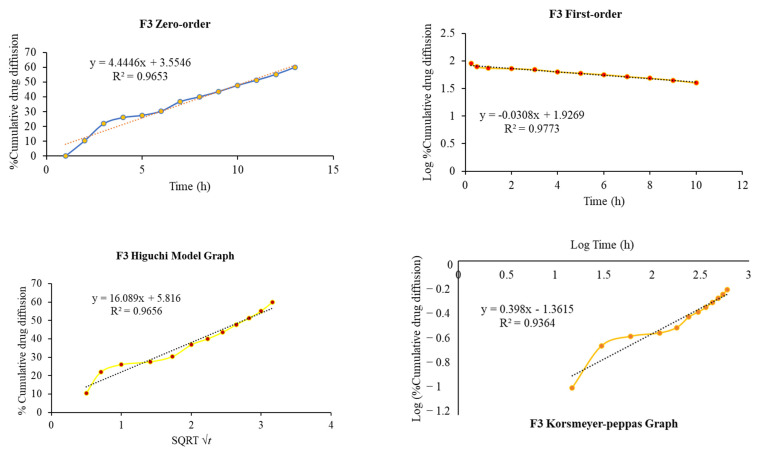
Different release kinetic models for in situ thermosensitive gel F3.

Table 4 and Figure 4 illustrate the correlation of dissolution data obtained for various release kinetic models, where all the formulations showed the different release kinetic models as evident by the highest regression (R^2^) values. The F1, F2, F3, and F4 formulations showed the Higuchi model, Korsmeyer–Peppas, first-order, and zero-order release kinetic models, respectively (Table 4). 

### 2.9. FTIR Analysis

**Figure 5:**
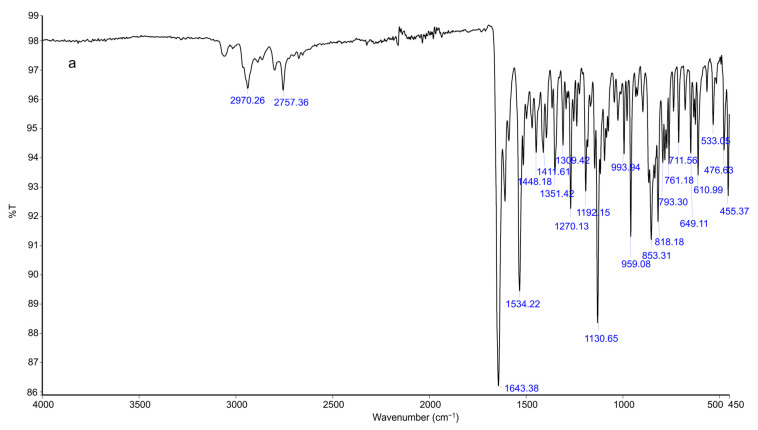

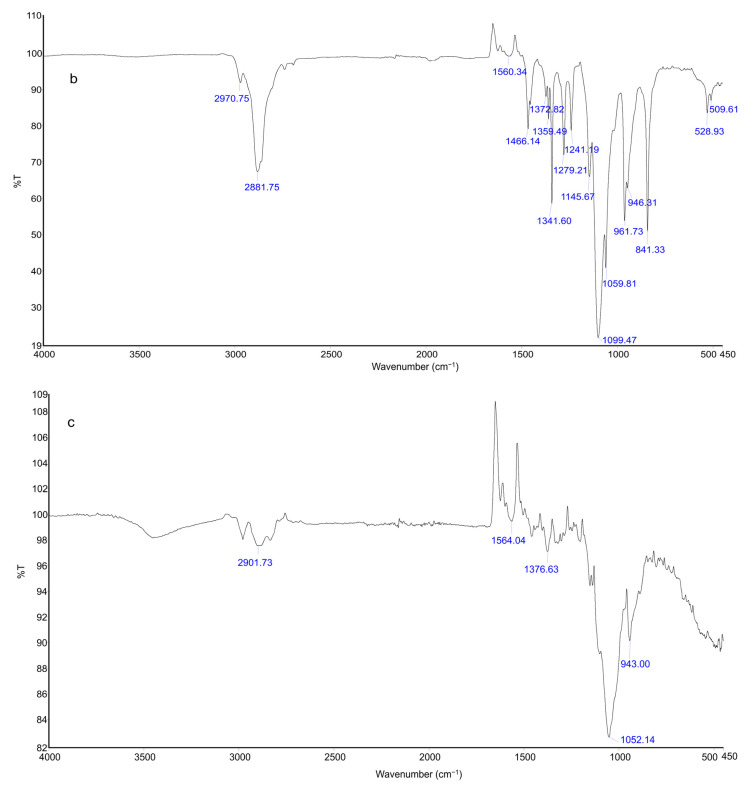

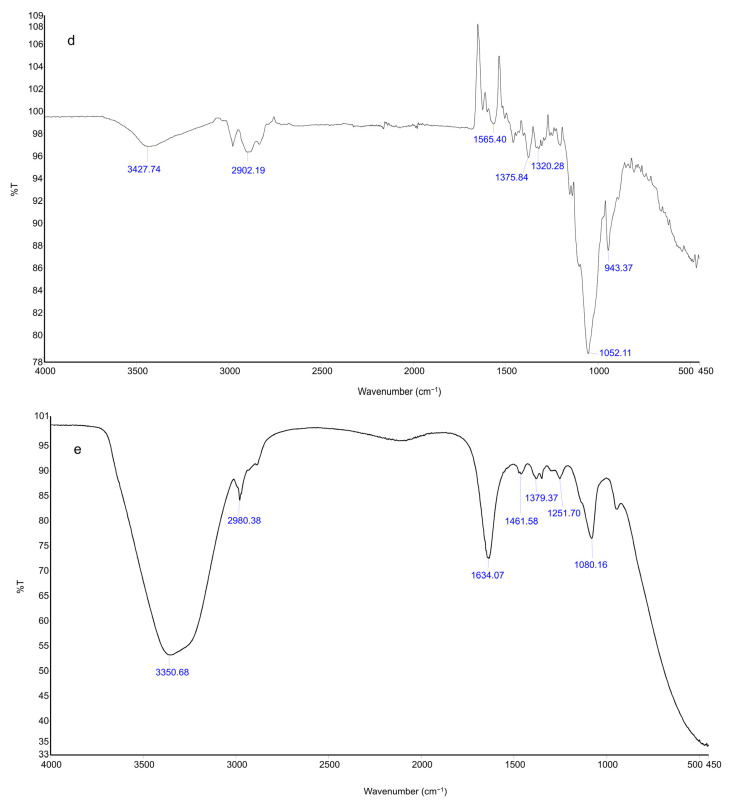
FTIR spectrum of (**a**) pure drug risperidone, (**b**) poloxamer 407, (**c**) HPMC K15M, (**d**) HPMC K100M, and (**e**) optimized in situ thermosensitive gel (F3).

To examine the compatibility of risperidone and the excipients used, an FTIR investigation was performed (Figure 5a–e). Spectrum bands of pure risperidone were at 2940.26 cm^−1^ and 2757.36 cm^−1^ due to aliphatic C–H stretching (Figure 5a). The other distinctive absorption bands were at 1643.38 cm^−1^ for the C=O stretching of aryl acids, 1534.22 cm^−1^ for the aromatic C=C group, 1448.18 cm^−1^ for the C=N stretching, 1411 cm^−1^ for the aliphatic C–H bending, 1351.42 cm^−1^ for the symmetric stretch of N-O group, 1130.65 cm^−1^ for the C–F stretching group, 1094.80 cm^−1^ for C-N stretching, and 959.08, 853.31, and 818.18 cm^−1^ due to the aromatic C–H bending group. Similar FTIR peaks were found as previously reported, confirming the purity of risperidone [2].

FTIR spectra of poloxamer 407 is represented by principal absorption peaks at 2970.75 cm^−1^ for CH_2_ stretch, 2881.75 cm^−1^ for aliphatic C-H stretch, 1359.49 cm^−1^ for in-plane O-H bend, 1130.65 cm^−1^ for C-O stretch, and 946.31 cm^−1^ for C-O-C linkage as shown in Figure 5b, similar to as formerly reported [42].

HPMC K15M (Figure 5c) showed the stretching of the alcohol C-O group at 1056.37 cm^−1^, an absorption band at 1376.63 cm^−1^ for the stretching of the C-O-ether linkage. It also showed the stretching of alkyl C-H at 2901.73 cm^−1^ and displayed similar peaks as previously reported [43].

The FTIR spectrum of HPMC K100M revealed the characteristic peaks at 3427.74 for OH stretching, 2902.19 for C-H stretching of alkanes, and 1052.75 for aliphatic C-O stretching (Figure 5d). It showed almost similar peaks to HPMC K15M polymer.

The FTIR spectrum of risperidone in the in situ thermosensitive gel was found to be entirely different from risperidone (Figure 5a). Some peaks of risperidone in the in situ thermosensitive gel (F3) either shifted, disappeared, or broadened (Figure 5e). The F3 formulation exhibited absorption bands at 1634.07 cm^−1^ for the C=O group, 1451.58 cm^−1^ for the C=N group, 1379.37 cm^−1^ for the N–O group, 1130.65 cm^−1^ for the C–F group, and 1080.16 cm^−1^ for C-N stretching. Other peaks in formulation showed a resemblance to poloxamer 407 and HPMC.

### 2.10. In Vivo Pharmacokinetics Studies

**Figure 6:**
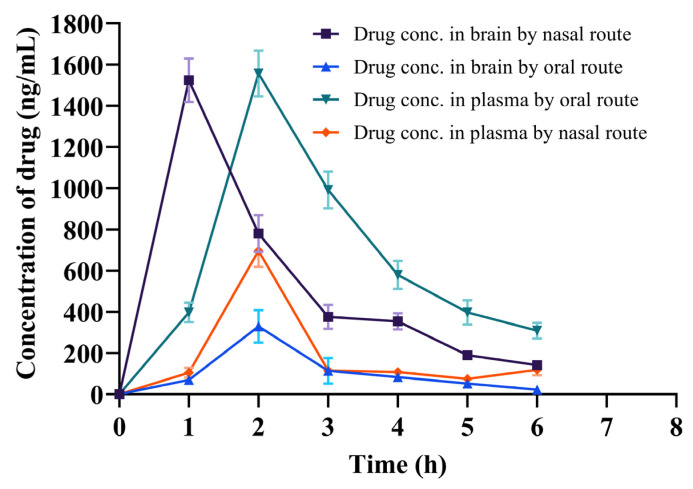
The brain drug concentration–time profile and plasma drug concentration–time profile of risperidone following oral and nasal (marketed and F3 in situ gel) administration in rats.

Nose-to-brain drug delivery platforms are a promising strategy for treating CNS disorders by bypassing the BBB and offering direct access to the brain. Because of the BBB, the dose concentration-time profile in the brain may differ significantly from that in the blood. Furthermore, therapeutically active compounds in the brain are distributed and eliminated via a variety of mechanisms, including diffusion, cerebrospinal fluid, and brain outer cellular fluid bulk flow, primary affinity for the target location, intracellular interchange, and nonspecific affinity components that are metabolized in brain tissues [44,45]. Also, the olfactory and trigeminal nerve pathways provide a direct connection between the nasal cavity and the CNS. Hence, drugs administered intranasally can reach the brain via these two primary routes [46,47]. Figure 6 and Table 5 demonstrate the risperidone brain drug concentration–time profile of a single dose after intranasal delivery of the F3 in situ gel and oral administration of the marketed tablet solution in rats.

The nasal and oral administration modes showed two different profiles (Figure 6). Table 5 explains that intranasal treatment confirmed *t*_max_ value (1 h), which was found to be lower than the oral route (2 h), hence suggesting that risperidone was quickly reached in the brain through the nasal route. Compared to oral administration of the marketed formulation, nasal route administration resulted in ~4.6X more C_max_ value (Table 5).

## 3. Discussion

The goal of the study was to develop an intranasal in situ thermosensitive delivery system that contains an antipsychotic drug by targeting the brain. The thermosensitive behavior of the in situ gel causes it to change phase from a liquid state at room temperature to a gel at physiological nasal temperature. Hence, the intended delivery system can deliver the drug to the brain to enhance patient compliance by preventing repetitive dosing.

HPMC is a conventional pharmaceutical excipient and semisynthetic derivative of cellulose and plays a crucial role in the advancement of pharmaceutical development [48]. It is essential for many therapeutic applications of mucoadhesive drug delivery systems due to its non-toxic and hydrophilic qualities. By adhering to the biological mucosa, it increases the continuity and tightness with the biological mucosa, further slowing drug release and increasing bioadhesion [49]. HPMC is also non-ionic, which means it does not interact with other ingredients or affect the pH of formulations, and is also non-toxic and safe in use.

Poloxamers are a family of triblock copolymers of poly(ethylene oxide)-b-poly(propylene oxide)-b-poly(ethylene oxide) (PEO-PPO-PEO), commonly known by their trade name Pluronic (also known as Synperonic or Kolliphor) [37]. Poloxamers are widely used in pharmaceutical and biomedical applications because of their amphiphilic nature, thermogelling behavior, and biocompatibility. Pharmaceutical researchers commonly use thermoresponsive poloxamers 407 and 188, according to the Food and Drug Administration (FDA).

Finding the ideal concentration ratio between poloxamer 407 and HPMC should be the first step in designing in situ thermosensitive formulations that could be administered into the nasal cavity and respond with a sol–gel transition at nasal temperature. Considering that, the formation of thermosensitive hydrogels with sufficient viscosity and partial rigidity, as well as the presence of the phase transition, requires a minimum concentration of 15–20 per cent of polymer [50]. It was observed that the change in the quantities of HPMC polymers resulted in the alteration of the in vitro properties of prepared in situ gel formulations. Also, poloxamer 407 polymer played a significant role in the sol–gel transition of in situ thermosensitive gel formulations under the influence of gelling temperature. Hence, poloxamer 407 was utilized for a thermoresponsive polymer, and HPMC was employed as a viscosity enhancer as well as a mucoadhesive agent in the development of in situ thermosensitive gels.

The pH of the nasal preparation is crucial for maintaining normal physiological ciliary movement, preventing the growth of harmful bacteria, allowing non-irritant adherence to the nasal mucosa, and leading to prolonged effect [51,52,53]. It is also reported that the normal nasal mucosa physiological pH is found in the range between 4.5 and 6.5, while the nasal mucosa can endure a pH range of between 3 and 10 of any nasal formulation [54]. Hence, it can be concluded that the formulated in situ thermosensitive gels show a pH range suitable for nasal delivery, as shown in Table 2.

It was observed that the gelation temperature and gelation time decreased as the concentration of HPMC increased (Table 2). Similar results were observed by the previous study [54]. This might be because, as the temperature rises, the internal energy of the poloxamer 407 breaks the hydrogen bonds between it and H_2_O [55,56]. As a result, the water molecules attached to the poloxamer chains are freed and migrate along with the spine. The mobility of free water molecules raises the entropy of the system, and to limit the change in entropy, hydrophobic chains seek to gather, and the solution form is later converted into the gel form.

The uniformity of drug content of any formulation is defined by the direct efficacy of the dosage form. The lowest drug content was found for F4, while the highest content was shown by the F3 formulation. The results of drug content (Table 2) indicate that the procedure utilized in the preparation of in situ thermosensitive gel was able to produce gels with minimum variability and uniform drug distribution.

Rheological characteristics should allow a formulation that is easy to spray, maintains a suitable viscosity for nasal residency, and is readily absorbed by the nasal mucosa to facilitate successful nasal drug administration. Therefore, to prolong residence time, formulation should be mucoadhesive and shear-thinning and have adequate viscosity. The optimum viscosity of various nasal gels lies in the range of 10^−1^ to 10^1^ Pa.s [37].

Therefore, the formulation needs to have a viscosity that allows for simple administration in solution form, followed by a quick sol–gel change at nasal temperature. With the obtained results, it was observed that HPMC K100M containing formulations showed high viscosity against HPMC K15M at both temperatures. The difference in the viscosity of formulations may be due to the percentage of HPC (8.5–10.5%) for HPMC K15M and HPC (9.5–11.5%) for HPMC K100M grades [57]. Furthermore, formulations became more viscous as the concentration of the mucoadhesive polymer increased. Such an effect is consistent with those observations reported in previous studies and can be linked to the increasing crosslinking of the polymers [52]. Viscosities decreased as the shear speed increased, as shown in Figure 2. The shear-thinning and non-Newtonian flow characteristics were observed in all thermosensitive gel formulations. Shear-thinning properties are thought to be advantageous for thermosensitive gels intended for nasal administration because they will improve the gels’ spreadability and retention at the application site [58].

To extend the gel’s nasal residency period, mucoadhesive strength is needed, because it is a crucial factor in the enhancement of the therapeutic effect. The F3 formulation showed the optimum mucoadhesive strength and viscosity, as reported previously [37,59]. Galgatte and colleagues reported the mucoadhesive strength of in situ gels in the range of 1550.73 to 738.50 dyne/cm^2^ and chose a mucoadhesive strength (738.50 dyne/cm^2^) suitable for in vivo studies [59]. All prepared formulations showed a remarkable variation in mucoadhesive force with the change in concentration and grade of HPMC (Table 3). HPMC exhibits limited interactions with drugs, predominantly through hydrogen bonding, but it plays a crucial role in supporting bioadhesion and enhancing localized drug delivery, as evidenced by higher retention rates [60,61]. Furthermore, the mucoadhesive strength may be achieved by the mucoadhesive polymer HPMC via the attachment of hydrophilic oxide groups with the oligosaccharide chain provided by poloxamer 407.

The release of the drug from the in situ thermosensitive gel depends on the viscosity of the developed formulation. The release pattern indicates that the thermosensitive in situ mucoadhesive intranasal gels have the ability to retain the drug in the matrix of the gel. Even at low concentrations of HPMC, the drug release was sustained, and it appeared to slow down as the concentration of HPMC increased. Also, the intranasal in situ thermosensitive formulation becomes a gel at the site of application, and drug release occurs from high to low concentration. Furthermore, the poloxamer 407 has the dual function of an amphiphilic surfactant and a polymeric carrier [62]. Poloxamer 407 can form micelles, distributing hydrophobic drugs into the micellar core [63,64], which results in improved drug solubility and dissolution profiles. Hence, the release profiles of risperidone were affected by both poloxamer 407 and the mucoadhesive polymer. The mucoadhesive polymer retarded the drug release from the in situ intranasal gel; the retarding effect of the mucoadhesive polymer can be ascribed to its capability to increase the formulation viscosity as well its ability to squeeze or distort the extra micellar aqueous channels of poloxamer micelles through which diffusion of the drug occurs, thus delaying the drug release process. As shown in Figure 5, in vitro drug diffusion from the F3 formulation follows the first-order release kinetics, indicating concentration-dependent drug diffusion from the in situ thermosensitive gel, with *n* < 0.45 (0.398) value indicating the Fickian-diffusion (diffusional case I).

The FTIR spectra of risperidone containing the in situ thermosensitive gel formulation demonstrated the weak interactions between the drug, HPMC, and poloxamer 407 (Figure 5e). This could be due to the formation of H-bonds or other weak bonds, for instance, van der Waals forces, as shown by the broadening of the peak. These interactions could be due to the formation of micelles by poloxamer 407 and the stabilization of their structures [63,64].

Following nasal delivery, it was found that the in vivo pharmacokinetic parameters were improved compared to those following oral administration (Table 5). This may be because the in situ gel of risperidone exhibited substantially better absorption; thus, it can improve bioavailability when administered intranasally, as indicated by a higher AUC0-∞ value (~5.4 times higher than the oral solution of the marketed formulation). Hence, it can be presumed that there is a higher rate of drug absorption from the in situ gel into the brain than from the oral route. The existence of BBB tight junctions is likely one factor contributing to the brain’s poorer absorption of oral drugs. Also, the increase in risperidone absorption from the designed in situ gel may potentially be because of better contact with the nasal membrane, to remain in the nostril for an extended period due to the presence of mucus, and to deliver the therapeutic active substance better [65,66].

Finally, it can be summarized that the absorption of risperidone in the brain was significantly (*p* < 0.05) increased when the formulated in situ gel was administered intranasally compared to the oral solution of the marketed formulation. According to these findings, intranasal administration of in situ gel increased the retention time and rate of drug penetration in the nasal cavity, thereby improving the absorption of risperidone in the brain compared to an oral solution of the marketed formulation.

## 4. Materials and Methods

### 4.1. Materials

Risperidone and Poloxamer 407 were purchased from Yarrow Chemicals Pvt. Ltd. (Mumbai, India). HPMC K100M and HPMC K15M were purchased from Otto Chemicals Pvt. Ltd. (Mumbai, India), and all other regents which were used for analysis were analytical and laboratory grade.

### 4.2. Formulation of Thermosensitive Mucoadhesive In Situ Gel

The thermosensitive mucoadhesive in situ gel was prepared by dissolving 180 mg of risperidone in 2.0 mL DCM using a magnetic stirrer with the required volume of phosphate buffer. Then 18% *w/v* poloxamer 407 solution was added under continuous stirring using a magnetic stirrer at a temperature below 10 °C using an ice bath. After that, the prepared solution was left for 24 h at 10 °C to obtain a clear and homogenous solution. Then, HPMC K100M and K15M at the concentration range of 0.3–0.5% *w/v* and benzalkonium chloride 0.1% *v/v* were added, and pH was adjusted up to 6.0 by utilizing 1N HCl or 1N NaOH solution to make various formulations (Table 1). Finally, the remaining volume of the phosphate buffer was added to make up the volume to 50.0 mL under continuous stirring and stored in amber-colored bottles until needed. The final concentration of the drug in the formulation was kept at 3.6 mg/mL.

### 4.3. Identification by FTIR

Fourier-transform infrared spectroscopy (FTIR) studies were implemented by utilizing a spectrometer (PerkinElmer, Norwalk, CT, USA). The test samples were formerly ground and blended carefully with potassium bromide (1 mg of sample to 80 mg of potassium bromide). Potassium bromide disks were made by compacting the powders using a hydraulic press at a pressure of approximately 60 Pascal. Scans were acquired at a resolution of 1 cm^−1^ from 4000 to 450 cm^−1^.

### 4.4. Appearance and pH Measurement

The clarity of prepared in situ gels was checked by visual examination. The pH of the developed formulations was measured by using a digital pH meter (Analab, Vadodara, India) in triplicate.

### 4.5. Gelation Time and Gelation Temperature

A 5.0 mL quantity of the in situ dosage form was transferred to a 10.0 mL test tube, which was then submerged in a thermoregulated water bath, and the temperature was increased slowly at a rate of 2 °C/min. The temperature taken to convert the solution into a gel-like structure was noted. Similarly, gelation time was measured by taking 5.0 mL formulation into a 10.0 mL test tube and putting the test tube in a pre-heated water bath at 37.0 °C, and the time required for the conversion of solution into gel was noted. The time and temperature for gelation were recorded in triplicate.

### 4.6. Drug Content Determination

In total, 1 mL of the formulation, equivalent to 3.60 g of drug, was weighed in a 250.0 mL volume flask. This was mixed with 100.0 mL of buffer solution for an hour on a magnetic stirrer, followed by a 20.0 min sonication to establish the perfect drug distribution. Absorbance of the solution was measured spectrophotometrically at λ_max_ of 280.0 nm by a UV spectrophotometer (Shimadzu UV-1800—UV-Vis, Kyoto, Japan) to calculate the drug amount. To determine content uniformity, the prepared in situ gel was assayed three times, and the drug content was calculated by using the regression equation (y = 0.014x − 0.0026), and the percentage of drug content was calculated by employing the following formula:

Drug content (%) = Conc. of drug in the test sample/equivalent conc. of drug taken × 100

### 4.7. Gelling Capacity and Spreadability

Gelling capacity was assessed by adding one drop of the prepared in situ gel formulation into a vial containing 2.0 mL freshly prepared simulated nasal fluid (pH 6.0) at 37 °C. The time taken for the conversion of formulation from solution to gel (sol to gel) and for gel to dissolve was examined visually. Gelling capacity was calculated in triplicate to measure the mean value and standard deviation.

Spreadability (S) was determined by an apparatus that consists of a wooden block, and provided by a pulley at one end, as previously reported [67]. A glass slide was fixed on this block. Spreadability was measured by placing the excess sample of the in situ gel between the two glass slides under a weight tension (1000 g) for 5 min to compress into a uniform thickness. A total of 50.0 g weight was added to the pan to pull off the upper slide. The time required to separate the two slides was noted. It is expressed in g. cm/s, calculated by using the following formula in triplicate: S = M × L/T, where M = weight tide to upper slide, L = length moved on the glass slide, and T = time taken.

### 4.8. Determination of Viscosity

The behavior of the sol–gel transition was further demonstrated by measuring viscosity at two different temperatures (10 °C and 35 °C) by using a Brookfield Viscometer (Model number DV-E 8661226, Stoughton, MA, USA) by utilizing spindle numbers 3 and 6. To evaluate the viscosity of the formulations, a 25.0 mL volume of the sample in a Nessler cylinder was taken. During measurement, the ramp speed was kept constant, while the angular velocity varied from 10 to 100 rpm with a holding time (10 s) at all speeds. Each sample’s viscosity was measured in triplicate.

### 4.9. In Vitro Mucoadhesive Strength

To assess each formulation’s mucoadhesive potential, the weight required to remove each dosage form from the nostril mucosal tissue was evaluated using a modified analytical balance methodology. Each glass vial was promptly sealed with a rubber band with a piece of nostril mucosa that was prepared from the goat nasal cavity (mucosal side out). Vials carrying nasal mucosa were kept at 37 °C for 5 min. A second vial holding a piece of mucosa was linked to the balance, while the first vial was put down on a pan with a height-adjustable stand, as shown in Figure 3. The nasal mucosa of the first vial was exposed to a set volume of samples from each formulation. The height of the second vial was then changed to bring the mucosal surfaces of the two vials near to one another, and 2 min of contact time was permitted to establish consistent contact between the tissues and the sample. The weight in the pan would increase until the vials came loose. The below equation was used to estimate the bioadhesive force, which was defined as the dissociation stress in dyne/cm^2^ based on the lowest weights required for tissues to separate from the interface of each in situ gel formulation.

Detachment stress (dyne/cm^2^) = M × g/A, where M is the weight needed to separate two vials in grams, g is gravitational acceleration (980 cm/s^2^), and A is the area of exposed tissue.

### 4.10. In Vitro Diffusion Study

Using an egg membrane, the in vitro diffusion of the drug from various in situ gel dosage forms was performed as per the previously reported method [54]. The egg membrane was prepared by keeping an empty egg cell in a 10% HCL solution until the outer hard layer was dissolved. Afterward, the membrane was thoroughly washed with distilled water three times. Then the egg membrane was fixed between the Franz diffusion cell donor and receptor compartments to check the permeation of the drug from a prepared gel. A simulated nasal buffer solution of pH 6.0 was filled as a permeation medium in the acceptor chamber. The temperature of the medium was maintained at 37 °C ± 0.5 °C with a stirring speed of 100 rpm. The donor chamber was filled with 1.0 mL of the prepared in situ gel formulation containing the drug. A 1.0 mL sample was withdrawn from the acceptor chamber at regular time intervals up to 12 h, and the replacement of the permeation medium of the same temperature. The sample was filtered through a 0.22μ filter and analyzed employing a UV–visible spectrophotometer at 280 nm to quantify the drug. The experiment was performed in triplicate.

### 4.11. In Vitro Drug Release Kinetics Modeling

To describe the drug release process, the cumulative quantity of risperidone released from the in situ gel formulations at various time intervals was fit into zero-order kinetics, first-order kinetics, the Higuchi model, and the Korsmeyer–Peppas model [68,69]. The goodness of fit was estimated by relating to the respective batches’ correlation coefficient (R^2^) values.

### 4.12. In Vivo Pharmacokinetics Studies

Before performing the animal study, the IAEC’s ethics committee approval at the Department of Pharmaceutical Sciences, KU, Uttarakhand, India, was obtained with study protocol number (KUDOPS/177/2023/07/22). The pharmacokinetic investigation was performed in 3 groups of Wistar albino rats (*n* = 6), each weighing between 250 and 280 g. Before starting the experiment, the rats were accustomed to seven days in polypropylene cages at ambient temperature, with adequate drinking water and animal food. Group 1 was kept under control and received only food and water. The oral RIS tablet suspension prepared with 0.5% CMC solution was given to group 2 by oral gavage, and the optimized F3 in situ gel was administered to group 3 at a dose of 0.09 mg/kg. At the end of the experiment, under light diethyl ether anesthesia at various time intervals (0.0, 1.0, 2.0, 4.0, 6.0, 8.0, 10.0 h), blood samples were drawn from the retro-orbital plexus in an EDTA-coated tube. Simultaneously, at each time interval, the animals were killed to collect their brains in an airtight container containing 10% formalin solution. The collected blood samples were centrifuged at 20 °C and 15,000 rpm for 10 min to collect the plasma. Furthermore, 400 µL of acetonitrile was added to 200 µL of plasma, and vortex mixing and centrifugation were performed to extract the drug. Also, the brain samples were homogenized in phosphate buffer and filtered to analyze the drug in the brain. The quantification of RS was performed by utilizing the previously reported HPLC-UV method [70] at 280 nm of the collected plasma and the homogenized brain supernatant samples. Using the PK Solver program, the pharmacokinetic parameters such as C_max_, T_max_, AUC, t_1/2_, and AUC_brain_/AUC_plasma_ were calculated.

### 4.13. Statistical Analysis

To analyze the statistical difference in results, the data were assessed by using SPSS 16.0 (SPSS Inc., Chicago, IL, USA, trial version). The data were calculated as the mean values with the standard deviation. Statistical analysis was performed using one-way ANOVA, followed by Dunnett’s test. A *p*-value < 0.05 was considered statistically significant.

## 5. Conclusions

Risperidone containing in situ thermosensitive hydrogel was successfully developed for nose-to-brain delivery; it exhibited sol–gel phase transition in response to temperature rise from storage temperature to body temperature and sustained drug release in buffer solution. Also, in situ gel showed suitable gelation temperature, sustained drug release, and enhanced intranasal absorption. In summary, this in situ gel system could provide a suitable platform for the sustained release of risperidone via the intranasal delivery. Hence, nose-to-brain dosing of risperidone can be an absolute alternative for an oral route. The in situ thermosensitive nasal gel formulation will also be an optimistic novel prescription employed in the clinical treatment of CNS disorders such as schizophrenia, with various advantages, for example, rapid absorption and onset of action, ease of application, high bioavailability, and self-administration. Further study is needed to assess the pharmacokinetics and bioavailability in humans, particularly the brain-targeting properties of risperidone in situ thermosensitive gel following the nasal route.

## Figures and Tables

**Table 1:** Compositions of the thermosensitive mucoadhesive in situ gel.

Name of Ingredients	Formulation Code			
	F1	F2	F3	F4
Risperidone (mg)	180.0	180.0	180.0	180.0
Poloxamer 407 (%*w*/*v*)	18.0	18.0	18.0	18.0
HPMC K100M (%*w*/*v*)	0.3	-	0.5	-
HPMC K15M (%*w*/*v*)	-	0.3	-	0.5
Dichloromethane (mL)	2.0	2.0	2.0	2.0
Phosphate buffer (mL)	q.s.	q.s.	q.s.	q.s.
Benzalkonium chloride (%*v*/*v*)	0.1	0.1	0.1	0.1

Note: The final volume was maintained up to 50 mL for all formulations; q.s. is quantity sufficient to make up the volume.

**Table 2:** Evaluated parameters of the in situ thermosensitive gels.

Formulation Code	Gelation Temperature (°C)	Gelation Time (Minutes)	Drug Content (%)	pH
F1	36.40 ± 0.95	2.05 ± 0.45	95.58 ± 2.37	6.37 ± 0.015
F2	37.50 ± 1.05	2.50 ± 0.55	97.12 ± 1.59	6.24 ± 0.011
F3	34.25 ± 1.10	1.65 ± 0.30	98.03 ± 1.68	6.25 ± 0.020
F4	35.80 ± 0.85	1.95 ± 0.60	96.14 ± 1.95	6.20 ± 0.026

(Where *n* = 3, values are presented as mean ± SD).

**Table 3:** Spreadability range, gelling capacity, and in vitro mucoadhesive strength of prepared thermosensitive mucoadhesive in situ gel (*n* = 3, mean = ±SD).

Formulation Code	Spreadability Range (gm/s)	Gelling Capacity (h)	In Vitro Mucoadhesive Strength (dyne/cm^2^)
F1	28.33 ± 1.52	9.13 ± 0.321	780.08 ± 3.92
F2	33.66 ± 2.08	8.35 ± 0.348	708.21 ± 4.52
F3	18.24 ± 2.15	9.52 ± 0.513	1110.65 ± 6.87
F4	27.41 ± 3.05	8.86 ± 0.264	943.41 ± 4.82

**Table 4:** Drug release regression (R^2^) values of formulations F1-F4.

Formulation Code	Zero-Order R^2^	First-Order R^2^	Higuchi R^2^	Korsymer–Peppas	
				R^2^	*n* Value
F1	0.988	0.979	0.994	0.990	0.827
F2	0.913	0.821	0.846	0.920	0.412
F3	0.965	0.977	0.965	0.936	0.398
F4	0.993	0.960	0.974	0.986	0.512

**Table 5:** Pharmacokinetic parameters of F3 and marketed formulation following oral and nasal route of administration (*n* = 6, mean ± SD).

Parameters	Brain		Blood	
Formulation	F3	Marketed	F3	Marketed
Route	Nasal	Oral	Nasal	Oral
*t*_max_ (h)	1.00	2.00	2.00	2.00
C_max_ (ng/g, ng/mL)	1523.56 ± 122.14 *	329.78 ± 35.08	696.13 ± 77.56	1556.32 ± 225.53
*t*_1/2_ (h)	1.49 ± 0.15	2.06 ± 0.19	2.08 ± 0.42	4.10 ± 1.03
AUC_0–∞_ (ng.h/mL)	4612 ± 316.53 *	848 ± 110.45	1425 ± 151.32	6432 ± 462.64
AUC_brain_/AUC_plasma_	3.236	0.132		

*t*_max_, time taken to obtain C_max_; C_max_, highest plasma/brain concentration of risperidone; AUC_0-∞_, area under the brain/plasma concentration–time profile from time 0 to ∞; AUC_brain_/AUC_plasma_, absolute bioavailability; t_1/2_, the half-life of the drug in plasma/brain (* *p* < 0.05 indicates a significant difference in results as compared to the marketed formulation).

## Data Availability

The data that support the findings of this study are available from the corresponding author upon reasonable request.
